# The Role of Enterobacteriaceae in Gut Microbiota Dysbiosis in Inflammatory Bowel Diseases

**DOI:** 10.3390/microorganisms9040697

**Published:** 2021-03-27

**Authors:** Valerio Baldelli, Franco Scaldaferri, Lorenza Putignani, Federica Del Chierico

**Affiliations:** 1 Multimodal Laboratory Medicine Research Area, Unit of Human Microbiome, Bambino Gesù Children′s Hospital, IRCCS, 00147 Rome, Italy; valerio.baldelli@opbg.net; 2 CEMAD, Unità Operativa Complessa di Medicina Interna e Gastroenterologia, Dipartimento di Scienze Mediche e Chirurgiche, Fondazione Policlinico Universitario “A. Gemelli” IRCCS, 00168 Rome, Italy; franco.scaldaferri@policlinicogemelli.it; 3 Dipartimento Universitario di Medicina e Chirurgia Traslazionale, Università Cattolica del Sacro Cuore, 00168 Rome, Italy; 4 Department of Diagnostic and Laboratory Medicine, Unit of Parasitology and Multimodal Laboratory Medicine Research Area, Unit of Human Microbiome, Bambino Gesù Children′s Hospital, IRCCS, 00165 Rome, Italy; lorenza.putignani@opbg.net

**Keywords:** Enterobacteriaceae, microbiota, inflammation, inflammatory bowel diseases (IBDs)

## Abstract

Inflammatory bowel diseases (IBDs) are a group of chronic gastrointestinal inflammatory diseases with unknown etiology. There is a combination of well documented factors in their pathogenesis, including intestinal microbiota dysbiosis. The symbiotic microbiota plays important functions in the host, and the loss of beneficial microbes could favor the expansion of microbial pathobionts. In particular, the bloom of potentially harmful Proteobacteria, especially Enterobacteriaceae, has been described as enhancing the inflammatory response, as observed in IBDs. Herein, we seek to investigate the contribution of Enterobacteriaceae to IBD pathogenesis whilst considering the continuous expansion of the literature and data. Despite the mechanism of their expansion still remaining unclear, their expansion could be correlated with the increase in nitrate and oxygen levels in the inflamed gut and with the bile acid dysmetabolism described in IBD patients. Furthermore, in several Enterobacteriaceae studies conducted at a species level, it has been suggested that some adherent-invasive *Escherichia coli* (AIEC) play an important role in IBD pathogenesis. Overall, this review highlights the pivotal role played by Enterobacteriaceae in gut dysbiosis associated with IBD pathogenesis and progression.

## 1. Introduction

Inflammatory bowel diseases (IBDs) are a heterogeneous group of chronic, relapsing–remitting, gastrointestinal (GI) inflammatory diseases with various degrees of damage, promoting the development of local and extra-intestinal complications. IBDs include ulcerative colitis (UC) disease and Crohn’s disease (CD); the first is restricted to the colon, while the second can affect different parts of the digestive tract [1,2].

Despite the etiology of IBDs remaining unknown, it is reported that there is an involvement of the host’s genetics, gut microbiota, and immune system [3,4,5]. Genetically susceptible individuals seem to be more prone to developing IBDs as a consequence of their atypical immune response to the autologous gut microbiota, following exposure to different environmental factors and stimuli [6,7]. Indeed, recent studies have revealed that pathologic alterations in the GI microbiota activate a mucosal immune response, thus developing chronic intestinal inflammation. These alterations in the GI microbiota trigger the so-called dysbiosis. Thus, the disruption of the intestinal eubiotic status can be considered a cause rather than simply a consequence of the chronic GI inflammation in IBDs [8,9,10,11,12].

**Figure 1:**
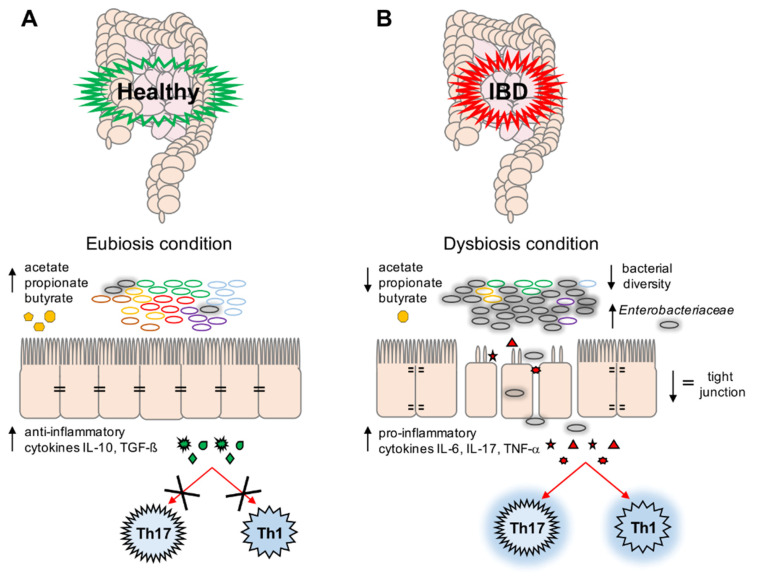
A schematic representation of eubiosis (**A**) and dysbiosis (**B**) conditions. (**A**) Microbiota plays an important role in the maintenance of gut stability by: (*i*) producing short-chain fatty acids (SCFAs) (i.e., acetate, propionate, and butyrate), (*ii*) preventing the expansion in any microbial pathogens, and (*iii*) modulating the immune system (i.e., production of anti-inflammatory cytokines (Interleukin (IL)-10, Transforming Growth Factor (TGF)-β)) and decreasing the activation of T-helper cell (Th)17 and Th1 cells; (**B**) During dysbiotic conditions, bacteria belonging to the Enterobacteriaceae family could overgrow, leading to a decrease in bacterial diversity and in gut stability. This is reflected by a decrease in SCFA production, and a parallel increase in pro-inflammatory cytokines such as IL-6, IL-17, and tumor necrosis factor (TNF)-α, which activate Th17 and Th1 cells involved in the inflammation response. Moreover, a decrease in tight junctions and a subsequent loss of impermeability in the intestinal epithelium is observed.

Many studies have reported an increase in the proportion of potentially harmful Proteobacteria, especially of the Enterobacteriaceae family, in IBD patients [2,13,14,15,16,17,18,19]. Therefore, it would appear that the host’s inflammatory response could be the trigger of gut microbiota imbalance, most likely caused by Enterobacteriaceae blooming, which leads to the persistence of IBD’s inflammatory state (Figure 1).

The main aim of this review is to describe and clarify the overgrowth of Enterobacteriaceae in IBDs, especially focusing on their role during the inflammation process in relation to metabolism changes observed in the gut microbiota of IBD patients. The secondary aim is to provide an update on certain adherent-invasive *Escherichia coli* (AIEC) strains frequently isolated from IBD patients, and their potential role in both CD and UC disease, discussing the difficulties in the characterization at species level of Enterobacteriaceae by targeted metagenomic approaches.

## 2. The Role of Gut Bacterial Microbiota in Inflammatory Bowel Diseases

Within the GI microbiota, the most studied component is the bacterial microbiota. Humans have more than 200 different bacterial species, with highly variable abundance in the gut. Thirty percent of this population is shared by different subjects, generating the core microbiota [20,21,22].

The bacterial microbiota plays important functions in the host, such as (*i*) the modulation of the immune system [23], (*ii*) the secretion of enzymes involved in the digestion of substrates not fully accessible to the host [24], and (*iii*) competition with pathogenic microorganisms for the same ecological niche [25]. In this context, the functionality of the GI tract is ensured and maintained also by the gut microbiota, which plays a positive role in the preservation of the intestinal barrier [26] (Figure 1A).

In particular, eubiotic microbiota is necessary to maintain the host’s immune homeostasis by the induction and function of T cells. Alterations in the gut microbiota’s composition can lead to the imbalance of T-cell subpopulations, such as Th1, Th2, Th17, and Treg cells, causing gut inflammation [27].

Numerous studies support the involvement of the GI microbiota in the pathogenesis of IBDs. Indeed, characteristic dysbiosis is often observed in subjects with CD and UC [28,29,30,31]; often, the induction or the maintenance of remission in IBDs is frequently related to the use of antibiotics and probiotics [32,33]. Several studies have demonstrated changes in microbial richness and evenness, and the relative abundance of specific bacterial taxa in IBD patients with respect to healthy subjects [34,35,36,37,38,39,40,41,42,43]. In particular, at the phylum level, the proportion of both Firmicutes and Bacteroidetes appears decreased, while the proportion of Proteobacteria and Actinobacteria is increased [13,44,45,46].

As shown by Geveres and co-workers, an increase in Veillonellaceae, Pasteurellaceae, Enterobacteriaceae, and Fusobacteriaceae, and a decrease in Bacteroidales, Erysipelotrichales, and Clostridiales, were correlated with IBDs’ clinical severity [38]. Similarly, a study on pediatric patients with CD concluded that the presence and severity of inflammation depends on microbiota dysbiosis; nonetheless, this study found an independent correlation between dysbiosis and other factors such as diet and/or the usage of antibiotics [39].

In particular, patients suffering from IBDs are associated with a decline in the abundance of protective anaerobic commensal bacteria, such as *Faecalibacterium prausnitzii*, *Clostridium* spp., and *Bacteroidetes fragilis*. *F*. *prausnitzii* has been shown to have anti-inflammatory properties, including the ability to modulate the host’s mucosal immune response by the production of short-chain fatty acids (SCFAs) [40]. SCFAs, such as acetate, propionate, and butyrate, are a primary energy source for colonic epithelial cells [40]. Thus, the underrepresentation of this bacterium in IBDs results in a decrease in the beneficial effects of SCFAs, including the inhibition of pro-inflammatory cytokine expression, the production of mucin and antimicrobial peptides, and tight junction protein downregulation [2,22,32,40,45,46] (Figure 1B).

Moreover, the gut microbiota in IBDs is often characterized by an expansion of translocating facultative aerobic bacteria—possible pathobionts—particularly belonging to the family of Enterobacteriaceae, such as *Escherichia* and *Shigella* [13,38,47,48,49]. Pathobionts are defined as commensal microorganisms that might become pathogens in the setting of a specific environmental stimulus in genetically susceptible subjects [50].

Another bacterium abundantly found in patients suffering from UC and CD is *Fusobacterium nucleatum* [51]. This bacterium showed the ability to adhere to and invade the intestinal barrier, inducing aberrant inflammation and exacerbating colitis, both in human and animal models [52,53,54,55,56]. Moreover, it has been recently proposed that the detection of *F. nucleatum* in the fecal metagenome could be used as an early biomarker of gut dysbiosis in IBDs and colorectal cancer [57].

## 3. The Role of Other Gut Microbiota Inhabitants in Inflammatory Bowel Diseases

Although fewer studies have examined the role of fungi and viruses specifically in the propagation of inflammation in IBDs, both of them are ubiquitous components of the intestinal microbiota [36]. As an example, patients with IBDs show differences in the presence of certain fungi compared with non-IBD controls, and different studies have revealed that this fungal dysbiosis is associated with an increased ratio between Basidiomycota and Ascomycota at the phylum level, indicating the emerging need to characterize the gut microbiota in IBDs [11,22,36,58,59,60,61].

Among viruses, bacteriophages are the predominant elements of the so-called virome in healthy humans [22,36]. In this context, changes in bacteriophage composition in IBD patients have been described [62,63,64]. However, whether bacteriophages could display a direct role in IBDs still remains unclear and has yet to be determined [36,65].

## 4. The Enterobacteriaceae Overgrowth in Inflammatory Bowel Diseases

These bacteria belonging to the Enterobacteriaceae family are localized near the intestinal mucosa due to their relatively higher tolerance of oxygen dispersed by the epithelium [66].

These bacteria are the first colonizers in the aerobic GI tracts of newborns. In fact, they are facultative anaerobes, which deplete oxygen to create a reduced environment suitable for following the colonization of strict anaerobes [67]. Following this, bacterial oligosaccharide fermenters, such as *Bifidobacterium*, bloom in the GI tract because of breast milk intake. Subsequently, the introduction of a solid food diet rich in polysaccharides produces the expansion of anaerobes and polysaccharide fermenters such as *Bacteroides*, *Clostridium*, and *Ruminococcus*, and a simultaneous decrease in *Bifidobacterium* and Enterobacteriaceae [68,69].

In IBD mouse models, microbiota dysbiosis in response to inflammation has been described, with a large relative increase in Enterobacteriaceae [48,70,71,72]. These studies support the notion that Enterobacteriaceae seem to have a growth advantage over other members of the gut microbiota commensals in the inflamed GI mucosa.

Moreover, an increment in Enterobacteriaceae was also associated with chronic UC inflammation compared to an acute status, showing a positive correlation of this bacterial family with a severe disease stage [73].

Furthermore, in a CD pediatric cohort, Enterobacteriaceae enrichment has been associated with an aggressive disease course with a higher risk of treatment failure [74].

In IBD patients, the functions of the gut microbiota have also been found to be perturbed. As an example, bacterial amino acid biosynthesis and carbohydrate metabolism are diminished, whereas nutrient uptake is enhanced [2]. In these patients, bacterial genes such as redox tolerance, secretion systems, and adherence and/or invasion, are overrepresented and the pathways linked to the production of bacterial SCFAs are repressed [37].

In a mouse model of UC, the blooming of Enterobacteriaceae has been linked to tetrathionate respiration, a metabolic pathway promoting intestinal colonization by *Salmonella enterica* subsp. *Typhimurium*, and with benzoate degradation, a pathway linked to Enterobacteriaceae growth and virulence pathways [75]. To date, the link between functional and compositional microbiota changes and IBD pathogenesis remains unclear. Moreover, it remains to be discovered whether the differences in the microbiota originating prior to the initiation of the disease process are a mere consequence of a hyperactive immune response.

The mechanisms by which Enterobacteriaceae bloom in IBDs, particularly during inflammation, are not completely known. In order to explain this observation, the “oxygen hypothesis” has been formulated [76].

In physiological conditions, enterocytes reduce oxygen levels in the gut lumen by beta-oxidation processes, creating an anaerobic environment [77]. In IBD patients in which intestinal inflammation occurs, beta-oxidation is reduced, and the oxygen level is increased. This event leads to an increase in facultative aerobes such as Proteobacteria/Enterobacteriaceae and, as a consequence, intestinal dysbiosis [78,79].

Moreover, in inflammatory conditions, an increment in nitrate production by the host has been demonstrated [80,81,82]. The expression of the gene encoding nitric oxide synthase is inhibited by the SCFA butyrate, via the activation of the peroxisome proliferator-activated receptor (PPAR) [83]. Butyrate represents the major energy source for enterocytes and it is involved in the maintenance of colonic mucosal health [84]. Intestinal inflammation leads to a reduction in healthy butyrate-producing microbiota, which leads to an increase in nitrate production and then the blooming of Enterobacteriaceae [83]. Consequently, the increased levels of both oxygen and nitrate observed in IBD patients could lead to Enterobacteriaceae overgrowth.

## 5. Inflammation and Enterobacteriaceae

Enterobacteriaceae contain molecular components that directly enhance the inflammatory response. Microbe-associated molecular patterns (MAMPs) are molecules located on the bacterial surface. These molecules interact with the receptors on immune cells to trigger inflammation [85]. One of the most potent MAMPs is the endotoxin lipopolysaccharide (LPS), which interacts with Toll-Like Receptor (TLR) 4 on immune cells [86]. The lipid-A of LPS can be acylated with five or six acyl chains. Hexa-acylated LPS shows immunostimulant activity that is 100-fold more active than penta-acylated LPS [87]. Enterobacteriaceae, and, in general, Proteobacteria, possess a more immunostimulatory version of LPS. Moreover, Enterobacteriaceae present unmethylated immunostimulatory motifs that can induce an immune response through an interaction with TLR-9 [88]. The blooming of Enterobacteriaceae, which contain these motifs, leads to a more selective pressure and shift toward an Enterobacteriaceae-dominated community [89].

Some scientific evidence leads to the hypothesis that dysregulation in innate immune systems induces Proteobacteria overgrowth, which promotes intestinal inflammation [90]. Regarding this, it has been reported that mice lacking TLR-5 developed transmissible spontaneous colitis associated with an abnormal expansion of Enterobacteriaceae [91]. Moreover, spontaneous colitis has also been reported in interleukin (IL)-10-deficient mice, due to their intolerance of intestinal microbiota. Indeed, IL-10 is the main anti-inflammatory cytokine required for immune tolerance to naïve microbiota [92], and in this mouse model, the onset and progression of gut inflammation has been correlated with an increase in *E. coli* [92].

Several studies also reported the role of Enterobacteriaceae in inducing the secretion of IL-8, tumor necrosis factor (TNF)-α, and IL-1β, and in the disruption of intestinal mucosa tight junctions, followed by an increment in intestinal permeability and inflammation [93,94].

Furthermore, a positive correlation between the nucleotide-binding oligomerization domain (NOD)-2 risk allele and Enterobacteriaceae was described in intestinal biopsies from an adult IBD patient cohort [95]. Since NOD-2 is an intracellular bacterial-sensing receptor that drives inflammatory signaling, this evidence strengthens the hypothesis of the direct involvement of Enterobacteriaceae in inflammation.

## 6. Bile Acid Dysmetabolism and Enterobacteriaceae in Inflammatory Bowel Diseases

In IBDs, an impairment in the microbial bile acid (BA) metabolism has been demonstrated, with high levels of fecal-conjugated BAs and low levels of secondary BA production [96]. These impairments in deconjugation and transformation abilities were associated with microbiomes [96].

In physiological conditions, cholesterol and BAs regulate multiple metabolic processes in the host [97,98]. Hepatocytes synthesize primary BAs from cholesterol conjugated to glycine and taurine. These conjugated BAs are stored in the gall bladder and are involved in lipid absorption and fat emulsification [99]. Gut microbiota carry out numerous BA biotransformation reactions [99], including the hydrolysis of conjugated BAs and glycine or taurine by bile salt hydrolase (BSH), and BAs’ 7α/7β-dehydroxylation by a multistep biochemical pathway found only in anaerobic bacteria [100].

An impairment in the gut microbiota’s metabolism significantly harms BAs’ metabolism and, consequently, the host’s glucose and cholesterol homeostasis [99]. Moreover, a decrease in BAs seems to favor the blooming of pathogenic and pro-inflammatory members of Enterobacteriaceae [101]. These changes in the gut microbiota’s ability of BA modification may be a significant factor in the onset or progression of IBDs [102]. Furthermore, modified BAs display altered binding profiles for BA receptors (i.e., Farnesoid X receptor (FXR)), inferring that the dysbiosis of the gut microbiota’s characteristic of IBDs may alter the capacity for BA modification in this community [103]. Cirrhotic patients have demonstrated a positive correlation between BA dysmetabolism and Enterobacteriaceae overgrowth [104]. Moreover, in obese patients the administration of vancomycin increased the abundance in Proteobacteria and, concomitantly, decreased fecal secondary BAs, with a simultaneous postprandial increase in primary BAs in plasma [105]. Finally, Heinken and co-workers demonstrated through a computational model that the dysbiotic IBDs’ microbiota were enriched in pathogenic *Escherichia* spp., which highly contributed to the BAs’ deconjugation transformation [106].

Future studies should be undertaken to validate and strengthen the evidence that Enterobacteriaceae play a significant role in BA dysmetabolism in IBDs, and to explore if BA modification driven by dysbiosis might represent a marker of IBDs’ onset or progression.

## 7. The Role of *Escherichia coli* in Inflammatory Bowel Diseases

One of the well-studied members of the Enterobacteriaceae family is *E. coli*, which is frequently able to colonize the human intestine [107]. This bacterium has been proposed as a possible cause of the beginning of disease in IBD patients [108]; indeed, several studies have found an increased number of virulent *E. coli* strains isolated from IBD patients with respect to healthy controls [108,109].

In the genome of *E. coli* strains, there are three nitrate reductases and three nitric oxide reductases. Hence, non-fermentable nitrates can be used by *E. coli* as a substrate for nitrate respiration and converted into fermentable nitrates. As a consequence, the expansion of *E. coli*/Enterobacteriaceae in the lumen of the inflamed gut observed in IBD subjects could reflect the high levels of non-fermentable nitrate generated in the gut by the host’s inflammatory response [80]. Consequently, in human and animal models, the presence of *E. coli* strains in IBDs has been reported (Table 1). However, whether *E. coli* strains may specifically benefit from nitrate respiration during IBDs has yet to be studied [94,110].

Most of the *E. coli* strains identified in IBD samples are AIEC [18,37,47,114,119]. AIEC members are (*i*) able to adhere to and invade epithelial cells utilizing a process dependent on actin microfilaments and microtubule recruitment; (*ii*) able to survive within the macrophage phagolysosome; (*iii*) able to induce the production of tumor necrosis factor (TNF) from infected macrophages; and, finally, lacking any defined invasive element [122,123]. The adaptive evolution of the genome of AIEC strains in a susceptible host and their ability to promote inflammation makes these microorganisms potential pathobionts [120,124,125,126].

Several studies have reported that different *E. coli* strains persist within epithelial cells and macrophages and selectively colonize the ileum of CD patients [111,112,113,114,120]. In particular, AIEC strains adhere to epithelial cells by the binding between the type 1 pili, expressed by bacteria, and the carcinoembryonic antigen-related cell adhesion molecule (CEACAM) 6, expressed on the apical surface of ileal epithelial cells [127]. Epithelial expression of CEACAM 6 is stimulated by interferon (IFN)-γ and TNF, which are overexpressed by macrophages harboring AIEC strains, generating a positive feedback loop [29,123].

*E. coli* strains from ileal biopsy from CD patients express several virulence genes mediating epithelial adherence and iron acquisition. Moreover, AIEC strains possess a wide arsenal of genes, such as *fliC*, *OmpC*, and others that regulate type 1 pilus expression and mediate epithelial cell adherence/invasion [29,128,129].

Further observations suggest that UC-associated AIEC could be distinct from those observed in CD, with greater invasive properties [115] or displaying different types of gene expression [121]. Although several studies confirmed the presence of AIEC strains in a higher proportion with respect to CD patients [116,118], others reported a lower prevalence compared with that of CD [115,117,119]. In the systematic review of Nadalian and co-workers, an increase in AIEC strains in UC patients emerged with respect to non-IBD controls, as well as the possible involvement of these bacterial strains in the pathogenesis of this disease [130].

Some studies of animal models suggest that the accumulation of AIEC and other *E. coli* strains in the gut could be a consequence of inflammation [17,72,92,131]. Moreover, several studies revealed that AIEC infection could induce changes in the gut microbiota [132,133]. Nowadays, it still remains unclear whether AIEC strains cause intestinal inflammation leading to IBDs or whether their blooming could be a consequence of inflammation, which leads to the aggravation of these diseases.

## 8. Identification of Enterobacteriaceae from Direct Samples

The number of genera and species in the Enterobacteriaceae family has markedly increased. Currently, this family comprises >60 genera and 250 species [134,135]. To better define the role of Enterobacteriaceae in gut inflammation or in intestinal microbiota dysbiosis, it is important to distinguish each family component at genus or species level. As reported in Table 1, the main studies in which Enterobacteriaceae species have been correlated to IBDs are based on bacterial identification using culture-based techniques. In fact, the low sequence variance in 16S rRNA partial gene sequencing, usually selected in 16S rRNA-based metagenomics, does not allow resolution at genus/species level [136,137].

Thus, it is mandatory to select other molecular targets that offer a superior discriminatory power of Enterobacteriaceae at the level of genus/species, especially for gut microbiota studies with targeted metagenomics approaches [138]. In the past, different genes, such as *tuf*, *atpD*, *dnaJ*, *rpoB*, *infB*, and *gyrB*, have been explored using molecular biology techniques [136,139,140,141,142].

Phylogenetic analyses of the *tuf* and *atpD* gene sequences have demonstrated controversial results in the identification of some Enterobacteriaceae species from isolated microbiological culture [136].

The *dnaJ* gene encodes the heat shock protein 40 and contains regions highly conserved in the Enterobacteriaceae family, suitable for broad-specificity primer design [143]. Despite this target demonstrating high performance in the unambiguous identification of Enterobacteriaceae species, it remains suboptimal for Enterobacteriaceae discrimination from direct specimens. In fact, the amplicon generated by polymerase chain reaction (PCR) is relatively long (758 base pair (bp)) for metagenomic-based approaches, which require short-length fragments [143].

The *rpoB* gene encodes the RNA polymerase subunit [139], and a partial region of this locus has been employed in describing nematode microbiota [144]. This study revealed that this target could be considered a highly appropriate marker for assessing the taxonomic structure of mock communities; however, further studies confirming this are required.

The *infB* gene is a universally distributed gene in all prokaryotes and encodes for translation initiation factor 2 (IF2) [140]. In Hedegaard and colleagues’ study, a partial sequence of *infB* was used to develop a method for identifying enterobacteria at species level. The authors concluded that *infB* can be used to both discriminate and classify strains of the same species [140].

The *gyrB* gene, which encodes the subunit B protein of DNA gyrase, has been previously used for describing phylogenetic relationships between Enterobacteriaceae [141]. A fragment of the *gyrB* gene (*gyrBint*) of 506 bp has been successfully employed to identify Enterobacteriaceae, proving its applicability in a clinical laboratory [145].

Nowadays, shotgun metagenomics approaches could allow gut microbiota taxonomic profiling, including Enterobacteriaceae, at species level, from direct samples [146,147,148]. However, sample contamination by high levels of the host’s DNA and the high cost of the analysis represent drawbacks of shotgun metagenomics with respect to targeted metagenomics [149]. Moreover, comparative studies on gut microbiota composition using both approaches have highlighted the differential depiction of bacterial profiles, although there was a consistent description of core microbiota [150,151]. Thus, both metagenomics approaches could be considered complementary in the genus-/species-level description of Enterobacteriaceae.

## 9. Concluding Remarks

There is a plethora of evidence on the contribution of microbiota dysbiosis to gut inflammation in IBDs. The loss of beneficial microbes that produce anti-inflammatory molecules favors Enterobacteriaceae as the environment for the expansion of pathogens and/or pathobionts. The proinflammatory nature of Enterobacteriaceae has been well established. These bacterial family members contain molecules such as MAMPs and LPS that directly enhance the inflammatory response of the host. Moreover, Enterobacteriaceae could also participate in intestinal dysmetabolism, altering the BA metabolism, formerly impaired in IBDs.

Amongst Enterobacteriaceae, AIEC strains colonize the intestinal mucosa of IBD patients. Several studies in animal models correlated the intestinal overgrowth of these bacteria to the gut inflammation trigger. However, the contribution of specific Enterobacteriaceae species or shifts in the IBD gut microbiota remains under debate. 

In fact, is emerged the difficulty to discriminate by the 16S rRNA-based metagenomics the effective composition of Enterobacteriaceae in IBDs and to define the active role of each component in these diseases.

The identification of Enterobacteriaceae components and the deduction of their function in intestinal dysbiosis in IBDs represents a stimulating challenge, requiring expertise and effort in order to improve the knowledge and the comprehension of the fine mechanism of IBDs’ pathogenesis and progression.

## Figures and Tables

**Table 1:** *Escherichia coli* strains identified in human and in animal models of inflammatory bowel diseases (IBDs).

Bacteria	Disease/Model	Microbiota Composition	Analysis Type	Reference
Adherent-invasive *E. coli* (AIEC)	IBD	Not investigated	Culture-based	[108]
*E. coli*	IBD	Not investigated	Culture-based	[109]
*E. coli* LF82	CD ^1^	Not investigated	Culture-based, PCR ^2^	[49]
*E. coli*	CD	↑Enterococci, *Bacteroides*	Culture-based	[111]
AIEC	CD	Not investigated	Culture-based, PCR	[112]
AIEC	CD	Not investigated	Culture-based, PCR	[113]
*E. coli*	CD	↑ *Klebsiella pneumoniae*, Bacteroidetes, ↓ Clostridia	16S rRNA sequencing	[14]
AIEC	CD	↑ Enterobacteriaceae ↓ Clostridiales	16S rRNA sequencing, Culture-based	[114]
AIEC	IBD	Not investigated	Culture-based, PCR	[115]
AIEC	IBD	Not investigated	Culture-based, PCR	[116]
*E. coli*	CD	↑ *Enterococcus* spp., *Clostridium difficile*, *Listeria* spp. ↓ *Faecalibacterium prausnitzii*	Microarray 16S rRNA	[46]
AIEC	CD	Not investigated	Culture-based, PCR	[117]
Entero-aggregative *E. coli* (EAEC) and AIEC	IBD	Not investigated	Culture-based, PCR	[118]
Enterobacteriaceae*/E. coli*	Induced colitis in T5KO ^3^ mice	↓ Bacteroidetes	16S rRNA sequencing	[91]
AIEC	IBD	Not investigated	Culture-based, PCR	[119]
Enterobacteriaceae*/E. coli*	Induced colitis in IL-10 -/- mice ^4^	↓ Bacteroidetes, Firmicutes, Verrucomicrobia	16S rRNA sequencing	[92]
AIEC	CD	Not investigated	Culture-based, PCR	[120]
*E. coli*	CD	↑ *Veillonella*, *Fusobacterium*, *Heamophilus* ↓ Lachnospiraceae, Ruminococcaceae, *Faecalibacterium*	16S rRNA sequencing	[38]
Diffusely adherent *E. coli* (DAEC) and AIEC	IBD	Not investigated	Culture-based, PCR	[121]
Enterobacteriaceae*/E. coli*	DSS-induced ^5^ colitis in C57BL/6 mice	↑ Clostridiaceae, Bacteroidaceae ↓ Lachnospiraceae, Lactobacillaceae	16S rRNA sequencing	[72]

^1^ Crohn’s Disease (CD); ^2^ Polymerase chain reaction (PCR); ^3^ Toll-like receptor 5 knockout (T5KO); ^4^ Interleukin (IL); ^5^ Dextran Sulfate Sodium (DSS).
